# Supplementary material for: Identification and Characterization of the BZR Transcription Factor Genes Family in Potato (Solanum tuberosum L.) and Their Expression Profiles in Response to Abiotic Stresses
Source: Plants (Basel). 2024 Jan 30;13(3):407. doi: 10.3390/plants13030407 (PMC10856970; doi:10.3390/plants13030407)
Supplement: Supplementary file 1 [file plants-13-00407-s001.zip › plants-2801303-supplementary/Supplementary Files/Figure S1. BZR amino acid sequence alignment of S. tuberosum.pdf]

```
                  *        20         *        40         *        60         *        80         *       100         *       120
StBZR3 : ----------------------------------------------MTSGTR---------------------QPTWKERENNKRRERRRRAIAAKIFAGLRMYGNYKLPKHCDNNEVLKALC :  56
StBZR2 : ----------------------------------------------MTAGTGGGGS-------------SGR-LPTWKERENNKRRERRRRAIAAKIFTGLRTQGNFKLPKHCDNNEVLKALC :  63
StBZR6 : ----------------------------------------------MTSGTR---------------------MPTWKERENNKRRERRRRAIAAKIFAGLRMYGNYQLPKHCDNNEVLKALC :  56
StBZR7 : MSNPHPHHHISNTQDPDPQFPNPNDDPNPNHPLQPQSHPQRRPRGFAATSAAA--------AGAGTINKSRKER-EKEKERTKLRERHRRAITSRMLAGLRQYGNFPLPVRADMNDVLAALA : 114
StBZR4 : ----------------------------------------------MTSGTR---------------------LPTWKERENNKRRERRRRAIAAKIFAGLRMYGNYKLPKHCDNNEVLKALC :  56
StBZR1 : MASEMQRYDTNEDDEEMGMDVKEEDDEDEDDEEKNAAMHAIAGFDGVHGSSSSNRFQHHQQYHEQPTPGGSRRCRPLEEKERTKLRERQRRAITAKILAGLRRHGNYNLRVRADINDVIAALA : 123
StBZR5 : ------------------------------MMWEGGGFPAEGGGGGEGGGGGGGS------------GSGRRKPSWRERENNRRRERRRRAMAAKIYSGLRAQGNYNLPKHCDNNEVLKALC :  81
StBZR8 : ------------------------------MMWEAGESPASSSAGAGAGGSGGAGVGI------PESGGGGRRKPSWRERENNRRRERRRRAVAAKIYTGLRAQGNYNLPKHCDNNEVLKALC :  87
                                                           g                        p w E4Enn4rRERrRRA6aa46  GLR  GN5 LpkhcDnNeV6kALc

               *       140         *       160         *       180         *       200         *       220         *       240
StBZR3 : KEAGWIVEEDGTTYRKGCKPVERMDIGG-SVSVSPCSSYQLS----------------PGASYNPSPVSSSIPSPISSHYVSNVQNNSDPNS-LIPWLKNLSS----GSSPSSSKFPHHLCIP : 157
StBZR2 : IEAGWIVEDDGTTYRKGYMPPP-IENGCASMNISACSSIQPS----------------PMSSSFPSPVPSYHASPTSSSFPSPSRCDGNPSSYILPFLHNLA------SIPST---LPPLRIS : 160
StBZR6 : NEAGWTVELDGTTYRKGCKPVERLDFLGGSTSLSPCSSYQPS----------------PFTSNNPSPASSSFPSPASSSYAANL--NMDGKS-LIPWLKNLSS----GSSSASSSKLPNFHIH : 156
StBZR7 : RQAGWTVEPDGTTFRQSPAPAPT-NASNMGTYQVMSVESPVS-----GSSFRNCSTRASVDGQPSVPRINESLSPAS--FDSIVVTESDTKVDKFTSTSPMNS---TGCLEAG-QLMQELHCG : 225
StBZR4 : KEAGWIVEEDGTTYKKGCKPVGHVDIIGYSASVSPCSSYQPS----------------PGASYNPSPASSSFPSPVSSNYVANVQNNHDPNT-LIPWLKNLSS----GSSPSSSRFPHHLYIP : 158
StBZR1 : REAGWVVLPDGTTFPSRSQPQGTGTAGGTPTTMVTSSSSHIPTQHTPPGSLKDVPSGFQNTDDQSACQMKSVFVPTSSPYDSSSTARSHTSAMVADGLDTQNDPFLVGSVDSIDKQVVDMHTK : 246
StBZR5 : VEAGWIVEPDGTTYRKGCRPTP-MEIGGTSANITPSSSRNPS----------------PPSSYFASPIPSYQPSPTSSSFPSPSRGDANMSSHPFAFLHS--------SIPLS---LPPLRIS : 176
StBZR8 : TEAGWIVEPDGTTYRKGCKPTP-MEIGGTSTNITPSSSRHPS----------------PPSSYFASPIPSYQPSPTSSSFPSPSRADANMSSHPYSFLHN--------VIPSS---LPPLRIS : 182
          2AGW Ve DGTT5 kg  P       g s      ss   s                p  s   sp  s   sP Ss 5 s               l          s            i

            *       260         *       280         *       300         *       320         *       340         *       360
StBZR3 : GG------SISAPVTPPLSS-------------------------------------------PTASTPRMNDNWENPTANSTWIQQHYPFLPYSTPPSPGRQTPPDSGWLSGVQTP-QDGPS : 230
StBZR2 : N---------SAPVTPPLSS-------------------------------------------PTR-GSKPPKPIWESLSRFPLHSFQHPLFAASAPSSPTRRRYSKPATIPECD-------E : 223
StBZR6 : TG------SISAPVTPPFSS-------------------------------------------PTARTPRIKT-------DAGWAGFRYPYLPSSTPASPGRQNFINAECFAGIS-----GPP : 218
StBZR7 : EHGSSFAETQYVPVFVMLSSGVINNFCQLMDPDGVKQELQQLKSLKIDGVVVNCWWGIVESWVPQKYEWSGYRELFKIIRDFNMKLQVVMAFHENGGSDTSGMFISLPQWVLEIGKDNQDIFF : 348
StBZR4 : GS------SISAPVTPPLSS-------------------------------------------PTARTPRMSD---DPMANSGWAQQHYAFLPSSTPASPGRQTPPDSGWLSGVQTP-QDGPS : 228
StBZR1 : LQERDFAGTPYIPVYVMLPLGVINMKSELVDADGLVKQLRVLKSINVDGVMVDCWWGIVEANAPQDYNWNGYKRLFQVVREHKLKIKVVMSFHECGGNIGDDVCIPLPHWVSEIGRSNPDIYF : 369
StBZR5 : N---------SAPVTPPLSS-------------------------------------------PTR-VPK-QIFNLETLARESMSALNIPFFAASAPTSPTRGQRFTPATIPECD-------E : 238
StBZR8 : N---------SAPVTPPLSS-------------------------------------------PTR-HPK-QTFNLETLAKESMFALNIPFFAASAPASPTRGQRFTPPTIPECD-------E : 244
                   saPVtpplss                                           Pt                             s p sp r

         *       380         *       400         *       420         *       440         *       460         *       480         *
StBZR3 : SPTFSLVSSNPFGFKEP-LSNGGSRMWTPGQSGTCSPAVGPC-----MDQTGDVP----MSDV-ISAEFAFGSNMKGVVKPWEGERIHEECISDDLELT--------------------LGNS : 322
StBZR2 : SDAVSVESARWVSFQTV----AAP---TSPTFNLVKPLPQQNILLDALSGHGMFGWGEAAAQKGHGAEFDFES--CKVK-AWEGERIHEVAV-DDLELT--------------------LGSA : 315
StBZR6 : SPTYSLVSPNPFGFKMDSLSRGGSRMCTPGQSGTCSPAIAAG-----LDRNADVP----MAEVMISDEFAFGSNIAGMVKPWEGERIHEDCVPDDLELT--------------------LGSS : 312
StBZR7 : TDRQGRRNTECLSWGIDKERVLRGRTAIEVYFDLMRSFRTEFD---DLFTDGLIS----AVEIGLGASGELKYPSFSERMGWRYPGIGEFQCYDKYSLQNLRKAATSRGHSFWAKGPDNAGYY : 464
StBZR4 : SPTFSLVSPNPFGFKEP-ISNGGSRMWTPGQSGACSPAIASG-----IDQTADVP----MSDA-ISAEFAFGSHMMGLVKPWEGERIHEECATDDLELT--------------------LGNS : 320
StBZR1 : TDRAGRRNPECLSWGIDKERVLRSRTAVEVYFDYMRSFRVEFD---EFFEDGIIS----MVEVGLGPCGELRYPSNPVKHGWRYPGVGEFQCYDQYLLKNLRKAAEARGHSFWARGPDNAGSY : 485
StBZR5 : SDSSTIDSGQWMSFQKY----AANGIPTSPTFNLIKPVAQR------IPSNDMI------IDKGKSIEFDFEN--VSVKAAWEGEKIHEVGL-DDLELT--------------------LGSG : 322
StBZR8 : SDSSTIDSGQWINFQKY----AAN-VPPSPTFNLVKPVPQP------LRPNDMI------TDKGKSIDFDFEN--VSVK-AWEGERIHDVGF-DDLELT--------------------LGSG : 326
         3      s     5                      p                               f f          Wege 6he    DdleLt                    lG

              500         *       520         *       540         *       560         *       580         *       600         *
StBZR3 : STR*----------------------------------------------------------------------------------------------------------------------- : 325
StBZR2 : KARA*---------------------------------------------------------------------------------------------------------------------- : 319
StBZR6 : KTR*----------------------------------------------------------------------------------------------------------------------- : 315
StBZR7 : NSKPHETGFFCERGDYDSYYGRFFLHWYRQVLIDHADNVLTLATLAFEGIQIVVKIPAIYWWYRTSSHAAELTAGYYNPTNQDGYSPVFEVLKKHSVTIKFVSSGLQVPETD----DALADPE : 583
StBZR4 : STR*----------------------------------------------------------------------------------------------------------------------- : 323
StBZR1 : NSRPQETGFFCDGGDYDGYYGRFFLNWYSQVMIDHADRVLSLAKLAFDGTCIAAKLSGIHWWYKTASHAAELTAGFYNPSNRDGYVAIAAMLKKHGAALNFKCAEMRMLEEAVDFCEALGDPE : 608
StBZR5 : TARM*---------------------------------------------------------------------------------------------------------------------- : 326
StBZR8 : NARI*---------------------------------------------------------------------------------------------------------------------- : 330
           4

           620         *       640         *       660         *       680         *       700
StBZR3 : ---------------------------------------------------------------------------------------- :   -
StBZR2 : ---------------------------------------------------------------------------------------- :   -
StBZR6 : ---------------------------------------------------------------------------------------- :   -
StBZR7 : GLSWQIMNSAWDKEINVAGNNAFPCYDREGFMRLVETAKPRNDPDRHRFSFFAFQQPSPLVQSAICFSELDYFIKCMHGEINNVES*- : 669
StBZR4 : ---------------------------------------------------------------------------------------- :   -
StBZR1 : GLAWQVLNAAWDVSLPVCSENALLCHDRGGYNCLLEKAKPLNDPDGKHIFAFTYLRLSPLLMDGQNYMEFERFVKRMHGEAVLEFPS* : 695
StBZR5 : ---------------------------------------------------------------------------------------- :   -
StBZR8 : ---------------------------------------------------------------------------------------- :   -
```
