# Supplementary material for: Identification and Characterization of the BZR Transcription Factor Genes Family in Potato (Solanum tuberosum L.) and Their Expression Profiles in Response to Abiotic Stresses
Source: Plants (Basel). 2024 Jan 30;13(3):407. doi: 10.3390/plants13030407 (PMC10856970; doi:10.3390/plants13030407)
Supplement: Supplementary file 1 [file plants-13-00407-s001.zip › plants-2801303-supplementary/Supplementary Files/Supplementary captions.pdf]

Figure S1. BZR amino acid sequence alignment of *S. tuberosum*. The BES1_N domain is boxed with light red color.

Figure S2. Syntenic relationships of the *BZR* genes within *S. tuberosum*. The black lines represent gene pairs.

Figure S3. Expression patterns of the eight *StBZR* genes under different spectrum. Different letters indicate significant differences between different tissues. Significant differences among the groups were compared based on Tukey's test ($P < 0.05$). The data points represent mean ± SD. The leaf development of the potato plantlets was severely inhibited under monochromatic red light, there was no leaf sample under red light.

Figure S4. Expression patterns of the eight *StBZR* genes after BR (50 μM, 0–24 h) treatment. Different letters indicate significant differences between different tissues. Significant differences among the groups were compared based on Tukey's test ($P < 0.05$). The data points represent mean ± SD.
