# Supplementary material for: Identification and Characterization of the BZR Transcription Factor Genes Family in Potato (Solanum tuberosum L.) and Their Expression Profiles in Response to Abiotic Stresses
Source: Plants (Basel). 2024 Jan 30;13(3):407. doi: 10.3390/plants13030407 (PMC10856970; doi:10.3390/plants13030407)
Supplement: Supplementary file 1 [file plants-13-00407-s001.zip › plants-2801303-supplementary/Supplementary Files/Table S1 Profiles of BZR gene family members of different species.pdf]

Table S1. Profiles of BZR gene family members of different species

Gene	Gene ID	Predicted Amino Acid Number/aa
AtBEH1	AT3G50750	276
AtBEH3	AT4G18890	284
AtBEH4	AT1G78700	325
AtBZR1	AT1G75080	336
AtBES1	AT1G19350	335
AtBEH2	AT4G36780	265
OsBZR1	OS07G0580500	298
OsBZR2	OS01G0203000	365
OsBZR3	OS06G0552300	355
OsBZR4	OS02G0233200	351
ZmBZR1	GRMZM5G812774	355
ZmBZR2	GRMZM5G852801	139
ZmBZR3	GRMZM2G307241	436
ZmBZR4	GRMZM2G446515	484
ZmBZR5	GRMZM2G069486	651
ZmBZR6	GRMZM5G868061	171
ZmBZR7	AC194970.5_FG002	316
ZmBZR8	GRMZM2G369018	363
ZmBZR9	GRMZM2G152172	378
ZmBZR10	GRMZM2G102514	317
ZmBZR11	GRMZM6G287292	345
NbBZR1	Niben101Scf00073	327
NbBZR2	Niben101Scf00219	330
NbBZR3	Niben101Scf00894	322
NbBZR4	Niben101Scf01983	329
NbBZR5	Niben101Scf03110	328
NbBZR6	Niben101Scf03282	240
NbBZR7	Niben101Scf03729	199
NbBZR8	Niben101Scf04132	328
NbBZR9	Niben101Scf05540	238
NbBZR10	Niben101Scf05948	325
NbBZR11	Niben101Scf06112	323
NbBZR12	Niben101Scf10412	231
NbBZR13	Niben101Scf10887	182
NbBZR14	Niben101Scf12841	316
SlBZR1	Solyc01g094580.3.1	703
SlBZR2	Solyc02g063010.3.1	322
SlBZR3	Solyc02g071990.3.1	328
SlBZR4	Solyc03g005990.3.1	327
SlBZR5	Solyc04g079980.3.1	332
SlBZR6	Solyc07g062260.3.1	318
SlBZR7	Solyc08g005780.4.1	674
SlBZR8	Solyc10g076390.2.1	182

SlBZR9	Solyc12g089040.3.1	337
