# Supplementary material for: Identification and Characterization of the BZR Transcription Factor Genes Family in Potato (Solanum tuberosum L.) and Their Expression Profiles in Response to Abiotic Stresses
Source: Plants (Basel). 2024 Jan 30;13(3):407. doi: 10.3390/plants13030407 (PMC10856970; doi:10.3390/plants13030407)
Supplement: Supplementary file 1 [file plants-13-00407-s001.zip › plants-2801303-supplementary/Supplementary Files/Table S2 Information of primer sequence.pdf]

Table S2. Information of primer sequence

Gene	Primer sequence (5′→3′)	GC (%)	Predicted Tm
StBZR1	*TGTCGATGGTGTGATGGTGG*	55	60.04
	AGTGAGGCAGTGGAATGCAA	50	59.89
StBZR2	*TCCGAAATCGGTCGAAGCAA*	50	60.04
	GTGCGGCTTCTCAAAACTCG	55	60.11
StBZR3	*GCTCCCGAAGCACTGTGATA*	55	59.82
	CGTTCAACTGGCTTGCATCC	55	60.11
StBZR4	*ATTCAGGGTGGGCACAACAA*	50	60.11
	GGTGTTTGACGACCAGGACT	55	59.89
StBZR5	*GATTGGAGGCACTTCAGCCA*	55	60.32
	CACGTGATGGACTGGGGAAA	55	59.96
StBZR6	*CTAGCCCTGGTCGTCAGAAC*	60	59.83
	CCAGGAGTGCACATTCGAGA	55	59.75
SBZR7	*TCCAAACCCGAATCACCCTC*	55	59.67
	AGCAAGCATTCGGCTCGTAA	50	60.39
StBZR8	*TGAAGCTGGTTGGATCGTTGA*	47.62	59.93
	TGCCGTGAAGAACTTGGTGT	50	60.11
EF-1-alpha	*GATGGTCAGACCCGTGAACA*	55	59.68
	CCTTGGAGTACTTCGGGGTG	60	59.75
