# Supplementary material for: Identification and Characterization of the BZR Transcription Factor Genes Family in Potato (Solanum tuberosum L.) and Their Expression Profiles in Response to Abiotic Stresses
Source: Plants (Basel). 2024 Jan 30;13(3):407. doi: 10.3390/plants13030407 (PMC10856970; doi:10.3390/plants13030407)
Supplement: Supplementary file 1 [file plants-13-00407-s001.zip › plants-2801303-supplementary/Supplementary Files/Table S3 Gene ID of syntenic genes in other species.pdf]

Table S3. Gene ID of syntenic genes in other species

Gene	Gene ID	Gene ID
StBZR1	Soltu.DM.01G033560	AT2G45880
StBZR1	Soltu.DM.01G033560	AT4G00490
StBZR1	Soltu.DM.01G033560	Solyc01g094580
StBZR1	Soltu.DM.01G033560	Nitab4.5_0000583g0040
StBZR2	Soltu.DM.02G006820	AT4G36780
StBZR2	Soltu.DM.02G006820	Solyc02g063010
StBZR2	Soltu.DM.02G006820	Nitab4.5_0000628g0120
StBZR3	Soltu.DM.02G015130	Os01t0203000
StBZR3	Soltu.DM.02G015130	Os02t0233200
StBZR3	Soltu.DM.02G015130	Os06t0552300
StBZR3	Soltu.DM.02G015130	AT4G18890
StBZR3	Soltu.DM.02G015130	Solyc02g071990
StBZR3	Soltu.DM.02G015130	Solyc03g005990
StBZR4	Soltu.DM.03G001120	AT4G18890
StBZR4	Soltu.DM.03G001120	Solyc02g071990
StBZR4	Soltu.DM.03G001120	Solyc03g005990
StBZR4	Soltu.DM.03G001120	Nitab4.5_0005428g0070
StBZR5	Soltu.DM.04G034930	AT1G19350
StBZR5	Soltu.DM.04G034930	AT1G75080
StBZR5	Soltu.DM.04G034930	AT4G36780
StBZR5	Soltu.DM.04G034930	Solyc04g079980
StBZR5	Soltu.DM.04G034930	Solyc12g089040
StBZR5	Soltu.DM.04G034930	Nitab4.5_0000110g0270
StBZR5	Soltu.DM.04G034930	Nitab4.5_0000790g0070
StBZR6	Soltu.DM.07G023410	Os01t0203000
StBZR6	Soltu.DM.07G023410	AT1G78700
StBZR6	Soltu.DM.07G023410	Solyc07g062260
StBZR6	Soltu.DM.07G023410	Nitab4.5_0000788g0010
StBZR7	Soltu.DM.08G003130	AT5G45300
StBZR7	Soltu.DM.08G003130	Solyc08g005780
StBZR8	Soltu.DM.12G005470	AT1G19350
StBZR8	Soltu.DM.12G005470	AT1G75080
StBZR8	Soltu.DM.12G005470	Solyc04g079980
StBZR8	Soltu.DM.12G005470	Solyc12g089040
StBZR8	Soltu.DM.12G005470	Nitab4.5_0000110g0270
StBZR8	Soltu.DM.12G005470	Nitab4.5_0000790g0070
