# Supplementary figures and images for: Identification and Characterization of the BZR Transcription Factor Genes Family in Potato (Solanum tuberosum L.) and Their Expression Profiles in Response to Abiotic Stresses
Source: Plants (Basel). 2024 Jan 30;13(3):407. doi: 10.3390/plants13030407 (PMC10856970; doi:10.3390/plants13030407)

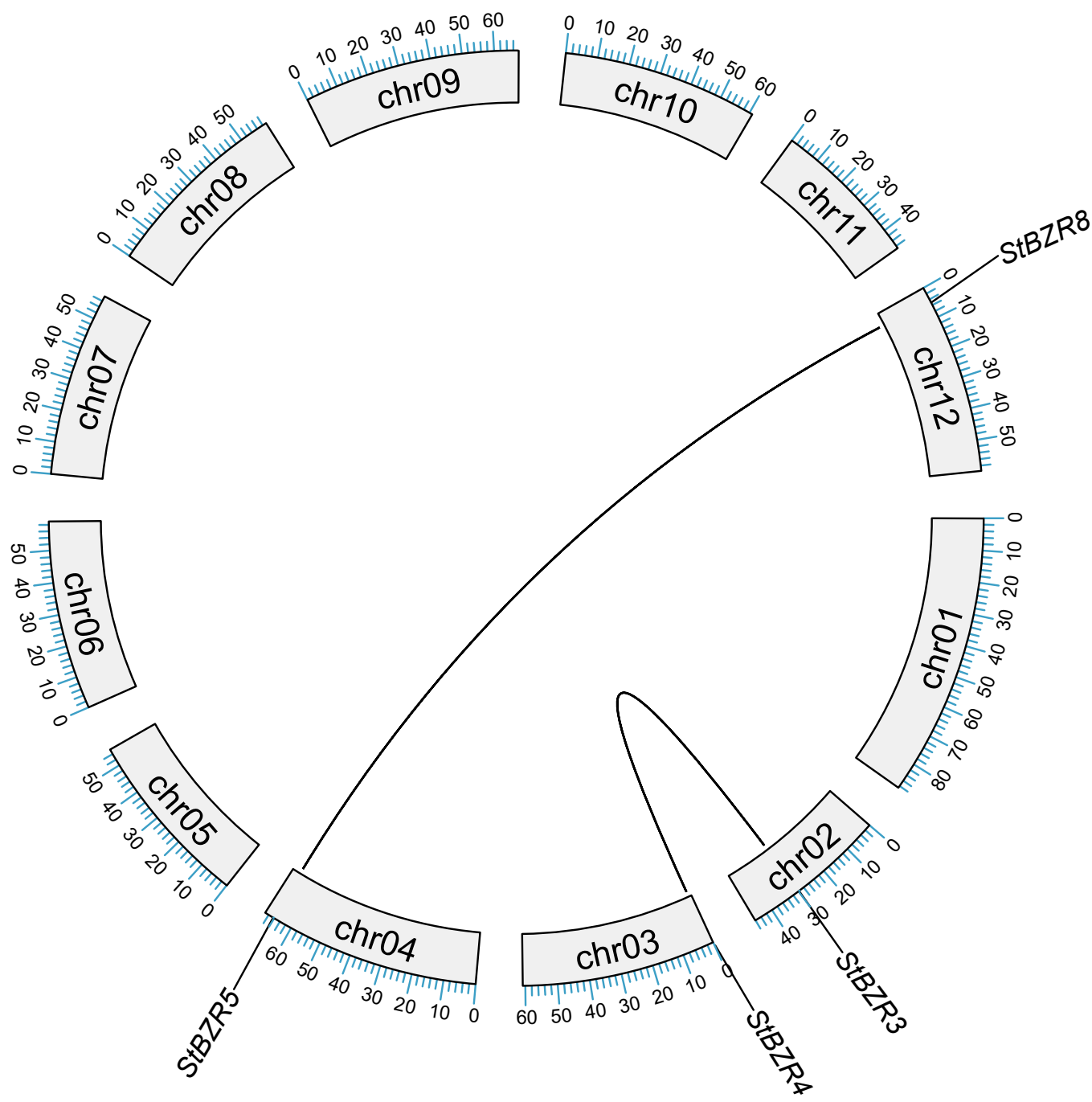

Supplement: Supplementary file 1 [file plants-13-00407-s001.zip › plants-2801303-supplementary/Supplementary Files/Figure S2. Syntenic relationships of the BZR genes within S. tuberosum.pdf]

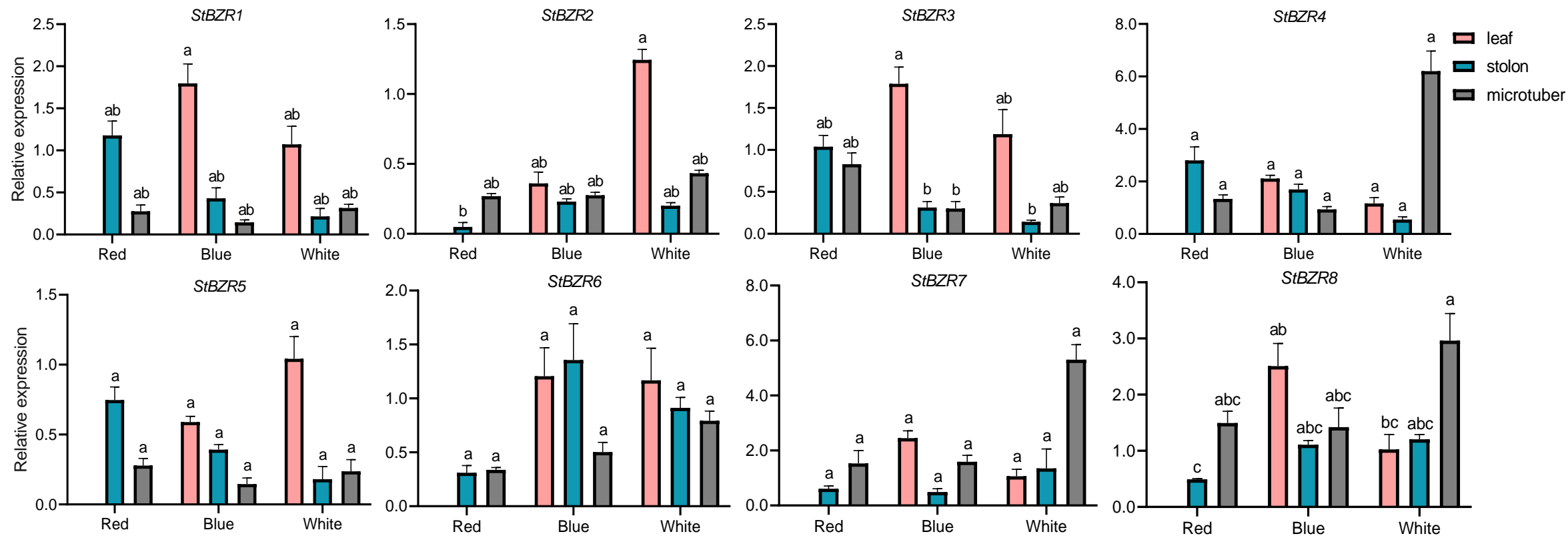

Supplement: Supplementary file 1 [file plants-13-00407-s001.zip › plants-2801303-supplementary/Supplementary Files/Figure S3. Expression patterns of the eight StBZR genes under different spectrum.pdf]

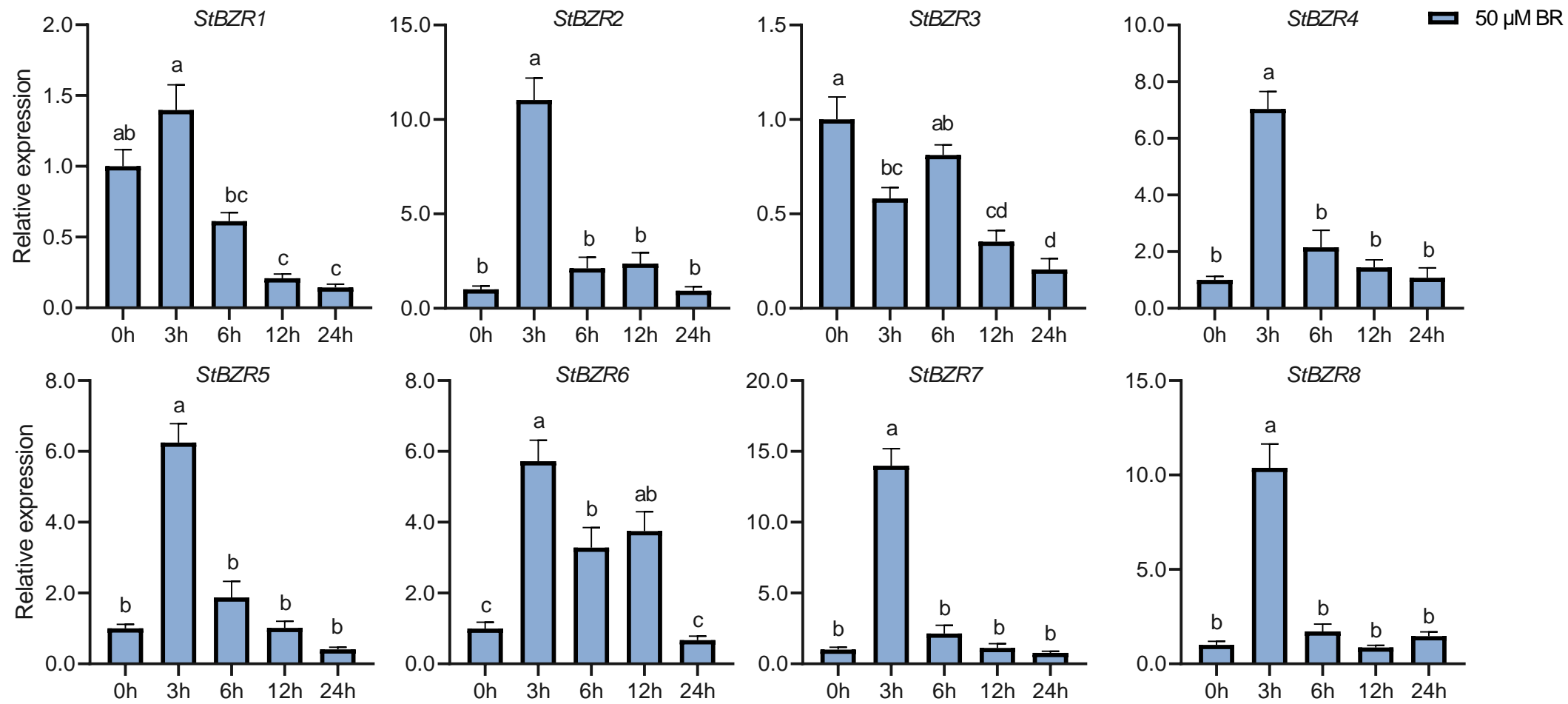

Supplement: Supplementary file 1 [file plants-13-00407-s001.zip › plants-2801303-supplementary/Supplementary Files/Figure S4. Expression patterns of the eight StBZR genes after BR (50 μM, 0–24 h) treatment.pdf]
